# General self-efficacy, self-confidence, and satisfaction in simulation-based nursing education: a structural equation modeling and mediation analysis

**DOI:** 10.3389/fmed.2026.1945224

**Published:** 2026-10-01

**Authors:** Majed M. Aljabri, Bandar S. Alharbi

**Affiliations:** Community and Psychiatric Mental Health Nursing Department, College of Nursing, King Saud University, Riyadh, Saudi Arabia

**Keywords:** general self-efficacy, mediation analysis, nursing education, self-confidence, simulation-based learning, structural equation modeling, student satisfaction

## Abstract

**Background:**

Simulation-based learning has become a fundamental component of nursing education, providing students with opportunities to develop clinical competence, critical thinking, and professional confidence in a safe learning environment. While learning satisfaction and self-confidence are recognized as key outcomes of simulation-based education, the extent to which general self-efficacy is associated with these outcomes remains insufficiently understood. This study examined the associations among general self-efficacy, self-confidence, and satisfaction with simulation-based learning among undergraduate nursing students.

**Methods:**

A cross-sectional study was conducted among undergraduate nursing students at King Saud University, Saudi Arabia. All enrolled nursing students were eligible to participate. A total of 209 complete responses were included in the final analysis. Satisfaction and self-confidence were measured using the Student Satisfaction and Self-Confidence in Learning Scale. General self-efficacy was assessed using the General Self-Efficacy Scale. Multiple linear regression identified factors associated with self-confidence and satisfaction, while structural equation modeling examined the hypothesized associations among latent constructs. Bootstrap mediation analysis was conducted to evaluate the indirect association between general self-efficacy and satisfaction through self-confidence.

**Results:**

Our findings showed that general self-efficacy was positively associated with self-confidence (*β* = 0.715, *p* < 0.001), and both general self-efficacy (*β* = 0.249, *p* = 0.002) and self-confidence (*β* = 0.650, *p* < 0.001) were positively associated with satisfaction. Students reporting a greater number of simulation sessions also demonstrated higher self-confidence (*β* = 0.352, *p* = 0.002), whereas academic year was inversely associated with self-confidence (*β* = −0.233, *p* = 0.037). Multiple linear regression produced findings consistent with the structural equation model. Mediation analysis showed a significant indirect association between general self-efficacy and satisfaction through simulation-related self-confidence (standardized indirect association = 0.465, *p* < 0.001), alongside a significant direct association (*β* = 0.249, *p* = 0.002).

**Conclusions:**

General self-efficacy and self-confidence were positively associated with nursing students’ satisfaction with simulation-based learning, with self-confidence demonstrating the strongest association and partially mediating the relationship between self-efficacy and satisfaction. Our findings highlight the importance of fostering students’ confidence and self-efficacy within simulation-based education. Because of the cross-sectional design, the observed relationships should be interpreted as associations rather than causal effects.

## 1 Introduction

Simulation-based education has become an integral component of contemporary nursing curricula (1, 2). This is because it provides students with opportunities to acquire and refine clinical knowledge, psychomotor skills, clinical reasoning, communication, and decision-making abilities in a safe, controlled, and learner-centered environment before engaging in direct patient care. By recreating realistic clinical scenarios without exposing patients to unnecessary risks, simulation facilitates experiential learning, encourages reflective practice, and enables repeated skill development through structured feedback and debriefing. Simulation-based learning has been increasingly adopted worldwide as an educational strategy to bridge the gap between theoretical knowledge and clinical practice while enhancing students' preparedness for complex healthcare environments (3).

Beyond the acquisition of technical competencies, simulation-based learning is intended to promote important psychological and educational outcomes that influence students' learning experiences and future clinical performance (4). Learning satisfaction and self-confidence are consistently recognized as key indicators of successful simulation experiences. Student satisfaction reflects learners' perceptions regarding the quality, usefulness, and educational value of simulation activities, whereas self-confidence represents students' beliefs in their ability to apply newly acquired knowledge and clinical skills effectively in practice. Higher levels of satisfaction and self-confidence have been associated with greater learner engagement, improved motivation, increased willingness to participate in future simulation activities, and enhanced readiness for clinical practice (3, 5). The interpretation of simulation-related outcomes should also be considered within the broader framework of established simulation pedagogy. The Healthcare Simulation Standards of Best Practice^TM^ developed by the International Nursing Association for Clinical Simulation and Learning (INACSL) emphasize the importance of clearly defined outcomes and objectives, appropriate simulation design, briefing, facilitation, debriefing, and evaluation in supporting effective simulation-based learning (6). These standards provide a disciplinary framework for understanding students' responses to simulation and are particularly relevant when examining outcomes such as self-confidence and satisfaction.

On the other hand, an individual's confidence during simulation-based learning, however, may extend beyond confidence gained from a specific educational activity. According to Bandura's Social Cognitive Theory, individuals' beliefs in their capability to organize and execute actions required to achieve desired outcomes, commonly referred to as self-efficacy, play a central role in determining motivation, persistence, resilience, and learning behaviors (7). Students with stronger general self-efficacy tend to approach challenging situations with greater confidence, demonstrate higher perseverance when confronted with difficulties, and actively engage in problem-solving activities (8). Within nursing education, these characteristics may facilitate more effective participation in simulation exercises.

Recently, empirical evidence suggests that general self-efficacy is positively associated with academic achievement, clinical competence, communication skills, and confidence among nursing students (9, 10). Similarly, studies have showed positive association between simulation experiences, student satisfaction, and self-confidence (11, 12). However, most previous investigations have primarily examined these variables independently or have focused on evaluating the effectiveness of simulation interventions, simulation design characteristics, or educational practices. However, the existing literature has largely examined general self-efficacy, simulation-related self-confidence, and satisfaction either independently or through pairwise associations. Less is known about how these constructs are simultaneously related when modeled as distinct latent variables, particularly whether simulation-related self-confidence accounts for part of the association between general self-efficacy and satisfaction (12). This represents an important gap because distinguishing the broader psychological construct of general self-efficacy from simulation-specific confidence may provide a more precise understanding of the factors associated with students' evaluations of simulation-based learning.

Importantly, general self-efficacy and simulation-related self-confidence represent related but distinct constructs. General self-efficacy reflects a broad belief in one's ability to cope with challenges and accomplish tasks across situations (7), whereas simulation-related self-confidence reflects perceived capability to apply knowledge, make clinical decisions, recognize learning needs, and perform nursing skills following simulation (13). From the perspective of Social Cognitive Theory, stronger general self-efficacy may influence students' engagement, persistence, and willingness to undertake challenging learning tasks (7). Within simulation-based learning, these characteristics may be associated with greater confidence in performing clinical tasks through opportunities for practice, feedback, and reflection. Previous study has also demonstrated positive associations between simulation-related self-confidence and satisfaction with simulation-based learning (14). Despite the increase in the implementation of simulation-based learning within nursing education in Saudi Arabia, evidence regarding the relationships among general self-efficacy, self-confidence, and satisfaction remains limited. Most available studies conducted in the region have focused on descriptive evaluations of simulation experiences or individual educational outcomes rather than examining the simultaneous associations among these constructs using robust multivariable analytical approaches. Consequently, evidence informing the psychological mechanisms underlying successful simulation experiences among Saudi nursing students remains scarce.

Based on Social Cognitive Theory and evidence from simulation-based nursing education, we hypothesized that general self-efficacy would be positively associated with simulation-related self-confidence, that both general self-efficacy and simulation-related self-confidence would be positively associated with satisfaction with simulation-based learning, and that simulation-related self-confidence would partially account for the association between general self-efficacy and satisfaction. These relationships were examined as theory-informed associations within the structural equation model rather than as causal pathways.

Therefore, we aimed to examine the associations among general self-efficacy, self-confidence, and satisfaction with simulation-based learning among undergraduate nursing students at King Saud University. Specifically, we aimed to (1) evaluate the measurement properties of the study instruments in the in our sample, (2) examine the associations between general self-efficacy, self-confidence, and satisfaction using structural equation modelling while accounting for measurement error, and (3) identify the associations of academic characteristics, including simulation exposure, academic year, and grade point average, with self-confidence and satisfaction using complementary multiple linear regression analyses. Given the cross-sectional design of the study, all estimated association are interpreted as associations rather than causal effects.

## 2 Methods

### 2.1 Study design and setting

We conducted a cross-sectional analysis among undergraduate nursing students enrolled at King Saud University (KSU), Riyadh, Saudi Arabia. KSU is one of the largest public universities in the Kingdom of Saudi Arabia and offers a comprehensive nursing curriculum that integrates classroom instruction with simulation-based clinical education. Simulation-based learning is routinely incorporated throughout the undergraduate nursing program to facilitate the development of clinical reasoning, psychomotor skills, decision-making, teamwork, and self-confidence in a safe and controlled educational environment before students undertake direct patient care. For this study, simulation sessions referred to scheduled simulation-based learning activities incorporated into the undergraduate nursing curriculum. The number of sessions represented students' cumulative exposure to simulation-based learning during their nursing education up to the time of survey completion. As simulation activities were integrated across different courses and academic years, the content and complexity of individual sessions could vary. Therefore, the number of sessions was considered an indicator of cumulative simulation exposure rather than a standardized measure of educational dose. The data collection was conducted from 8 June 2026 to 22 June 2026. During this period, nursing students were invited to participate in an anonymous online survey evaluating their perceptions of simulation-based learning, general self-efficacy, and self-confidence.

### 2.2 Participants and recruitment

The target population are all undergraduate nursing students registered in the College of Nursing at King Saud University during the study period. Because the objective was to obtain perspectives from students with diverse academic backgrounds and varying levels of exposure to simulation-based education, all currently enrolled nursing students were considered eligible for participation, regardless of academic year or cumulative grade point average (GPA). Participation was voluntary. Students were informed that participation or non-participation would have no influence on their academic evaluation, grades, or relationship with faculty members. Recruitment was undertaken using an institutional electronic announcement strategy. An invitation describing the purpose of the study, eligibility criteria, voluntary nature of participation, confidentiality assurances, and a Quick Response (QR) code linking directly to the online questionnaire was disseminated through official student announcement boards across the university. Students accessed the questionnaire by scanning the QR code using their personal mobile devices. This recruitment strategy has been successfully employed in several previous survey-based studies conducted within the university and enabled broad dissemination while minimizing researcher influence during participant recruitment.

A total of 289 students accessed and submitted the online questionnaire during the data collection period. Following data screening, 80 responses were excluded because of missing data across several study variables, particularly individual items of the study instruments. The remaining 209 responses contained complete data for the demographic characteristics and all questionnaire items and were retained for the main analyses. Missing data were limited to the number of simulation sessions attended, which was unavailable for seven participants (3.3%), resulting in 202 participants with valid data for this variable. No questionnaire items measuring satisfaction, self-confidence, or general self-efficacy had missing values. The SEM and regression models including simulation-session exposure were therefore estimated using 202 participants.

### 2.3 Study measures

Three validated self-report instruments were used to assess students' perceptions of simulation-based learning, self-confidence, and general self-efficacy. All questionnaires have been widely used in nursing education research and demonstrate satisfactory psychometric properties across diverse educational settings.

Students' satisfaction with simulation-based learning was measured using the Satisfaction subscale of the Student Satisfaction and Self-Confidence in Learning Scale (SSSCL) developed by the National League for Nursing (NLN) (15). The satisfaction subscale comprises five items designed to evaluate learners' perceptions regarding the effectiveness, usefulness, and overall quality of simulation experiences. Representative items assess whether simulation activities provide meaningful learning opportunities, enhance understanding of clinical concepts, and meet students' educational expectations. Responses were recorded using a five-point Likert scale ranging from 1 (strongly disagree) to 5 (strongly agree), with higher scores indicating greater satisfaction with simulation-based learning. The original English-language instrument was used without translation or language adaptation. The SSSCL was developed for evaluating students' perceptions of satisfaction and self-confidence following simulation-based learning experiences The SSSCL has demonstrated strong psychometric performance in nursing education research across different countries, with reported Cronbach's alpha coefficients generally exceeding 0.80 (16, 17).

Self-confidence associated with simulation-based learning was assessed using the Self-confidence subscale of the Student Satisfaction and Self-Confidence in Learning Scale (16), which consists of eight items measuring students' confidence in applying clinical knowledge, making clinical decisions, recognizing learning needs, and performing nursing skills following simulation experiences. Each item was rated on the same five-point Likert scale ranging from 1 (strongly disagree) to 5 (strongly agree). Higher scores reflected greater confidence in learning and performing clinical activities following simulation exercises. Our study showed excellent internal consistency.

General self-efficacy was measured using the General Self-Efficacy Scale (GSES) developed by Schwarzer and Jerusalem (18). The original English-language version was administered without translation or language adaptation. The instrument consists of 10 items designed to evaluate individuals' overall confidence in their ability to successfully cope with difficult situations, overcome challenges, and accomplish goals through personal effort and adaptive problem-solving. Participants rated each statement using a four-point Likert scale ranging from 1 (not at all true) to 4 (exactly true). Item scores were summed to generate an overall self-efficacy score, with higher scores representing greater perceived general self-efficacy. The GSES has been translated into numerous languages and extensively validated across different cultural and educational contexts, including healthcare education.

### 2.4 Internal consistency and convergent validity

Before testing the hypothesized structural relationships, we evaluated the psychometric properties of the measurement model. Internal consistency reliability was assessed using both Cronbach's alpha (*α*) and McDonald's omega (*ω*). Convergent validity was evaluated using the average variance extracted (AVE), with values of 0.50 or greater considered indicative of adequate convergence between latent constructs and their observed indicators. Confirmatory factor analysis (CFA) was subsequently conducted to evaluate construct validity and verify the adequacy of the hypothesized three-factor measurement model comprising satisfaction, self-confidence, and general self-efficacy.

Model fit was evaluated using multiple complementary goodness-of-fit indices, including the Comparative Fit Index (CFI), Tucker–Lewis Index (TLI), Root Mean Square Error of Approximation (RMSEA) with its 90% confidence interval, Standardized Root Mean Square Residual (SRMR), and the chi-square statistic. Consistent with established recommendations, values of CFI and TLI ≥ 0.90 (preferably ≥ 0.95), RMSEA ≤ 0.08, and SRMR ≤ 0.08 were considered indicative of acceptable model fit.

### 2.5 Statistical analysis

Descriptive statistics were calculated to summarize participants' demographic characteristics. Continuous variables are presented as means and standard deviations, whereas categorical variables are summarized using frequencies and percentages. Pearson correlation coefficients were calculated to examine the bivariate relationships among satisfaction, self-confidence, and general self-efficacy.

Multiple linear regression was used as a complementary analysis to examine factors associated with satisfaction with simulation-based learning. The explanatory variables used in the model were general self-efficacy, number of simulation sessions attended, cumulative grade point average (GPA), academic year, age, gender, reason for choosing nursing, and simulation-related self-confidence. Regression coefficients were reported as unstandardized B values with standard errors, 95% confidence intervals (CIs), and *p* values. The regression analysis was used to examine the independent associations of these variables with satisfaction before evaluating the hypothesized relationships among the latent constructs using structural equation modeling.

Structural equation modeling (SEM) was subsequently performed to simultaneously evaluate the measurement model and the hypothesized structural relationships while accounting for measurement error. The structural equation model retained the three-factor measurement model specified in the confirmatory factor analysis, including the same correlated residuals. In the structural component, simulation-related self-confidence was regressed on general self-efficacy, number of simulation sessions attended, GPA, and academic year. Satisfaction with simulation-based learning was regressed on simulation-related self-confidence, general self-efficacy, number of simulation sessions attended, GPA, and academic year. The three observed covariates were included as exogenous predictors. Model parameters were estimated using the weighted least squares mean- and variance-adjusted (WLSMV) estimator, and standardized path coefficients (*β*), standard errors, *z*-statistics, and corresponding *p*-values were reported. Because the study was cross-sectional, these structural paths represent theory-driven associations rather than causal effects, and the estimated coefficients should be interpreted as measures of association between the latent constructs.

For the CFA and SEM, questionnaire items were treated as ordered categorical indicators., and model parameters were estimated using WLSMV estimator. This approach was selected to account for the ordinal response format of the four-point Generalized Self-Efficacy Scale and five-point Student Satisfaction and Self-Confidence in Learning items. Model fit was evaluated using multiple complementary goodness-of-fit indices, including the chi-square statistic (*χ*^2^), Comparative Fit Index (CFI), Tucker–Lewis Index (TLI), Root Mean Square Error of Approximation (RMSEA) with its 90% confidence interval, and the Standardized Root Mean Square Residual (SRMR). Consistent with established recommendations, CFI and TLI values ≥ 0.90 (preferably ≥ 0.95), RMSEA values ≤ 0.08, and SRMR values ≤ 0.08 were considered indicative of acceptable model fit.

Discriminant validity was assessed using the heterotrait-monotrait ratio (HTMT), calculated from the polychoric correlation matrix of the ordinal questionnaire items. HTMT values were examined against conventional thresholds, with values below 0.85 indicating stronger evidence of discriminant validity and values approaching 0.90 considered indicative of substantial construct overlap.

To examine whether self-confidence mediated the association between general self-efficacy and satisfaction, mediation analysis was conducted within the SEM framework using bias-corrected bootstrap resampling with 5,000 bootstrap samples. The average causal mediation effect (ACME), average direct effect (ADE), total effect, and proportion mediated were estimated together with their corresponding 95% bootstrap confidence intervals. Statistical significance was defined as a two-sided *p*-value < 0.05. Because our study used a cross-sectional design, these quantities were interpreted as statistical associations within the specified mediation model and not as evidence of causal or temporal mediation.

## 3 Results

In this study, there were a total of 209 undergraduate nursing students participants (Table 1). More than half were aged 21–23 years (56.5%), while 28.7% were aged 24–26 years and 14.8% were aged 18–20 years. 62.2% of the participants were female and 32.2% are in their 3rd year, followed by second-year students (23.4%), fourth-year students (16.3%), internship students (14.8%), and first-year students (13.4%). Most participants had a grade point average (GPA) between 3.50 and 4.49 (54.5%), whereas 26.3% had a GPA of 4.50–5.00 and 19.1% had a GPA below 3.50. Nearly half of the students (45.5%) had attended one to two simulation sessions, while 33.0% had attended three to five sessions, 18.2% had participated in more than five sessions, and 7 (3.3%) are missing. Employment or career prospects were the most frequently reported reason for choosing nursing (55.5%). Participants reported a moderate-to-high level of satisfaction with simulation-based learning (mean = 3.30, SD = 0.86) and a moderate level of self-confidence (mean = 3.15, SD = 0.83), based on a 5-point response scale. The mean general self-efficacy score was 25.7 (SD = 5.2) on a scale ranging from 10 to 40.

**Table 1:** Characteristics of the study participants and descriptive statistics of study variables (*N* = 209).

Characteristic	*n* (%) or Mean ± SD
Age group (years)	
18–20	31 (14.8)
21–23	118 (56.5)
24–26	60 (28.7)
Gender	
Female	130 (62.2)
Male	79 (37.8)
Academic year	
First year	28 (13.4)
Second year	49 (23.4)
Third year	67 (32.1)
Fourth year	34 (16.3)
Internship	31 (14.8)
Grade point average (GPA)	
<3.50	40 (19.1)
3.50–4.49	114 (54.5)
4.50–5.00	55 (26.3)
Simulation sessions attended	
1–2	95 (45.5)
3–5	69 (33.0)
>5	38 (18.2)
Missing	7 (3.3)
Primary reason for choosing nursing	
Employment/Career	116 (55.5)
Personal motivation	72 (34.4)
External influence	21 (10.0)
Study variables	
Satisfaction	3.30 ± 0.86
Self-confidence	3.15 ± 0.83
General self-efficacy	25.7 ± 5.2

The measurement scales demonstrated acceptable to excellent internal consistency (Supplementary Table S1). Cronbach's *α* ranged from 0.77 to 0.92 and McDonald's *ω* ranged from 0.73 to 0.91. Satisfaction (AVE = 0.70) and self-confidence (AVE = 0.65) exhibited adequate convergent validity (AVE ≥ 0.50), whereas the general self-efficacy scale showed lower convergent validity (AVE = 0.30), despite maintaining acceptable internal consistency.

A study variables were positively and significantly correlated (*p* < 0.001) (Supplementary Table S2). Satisfaction showed a strong positive correlation with self-confidence (*r* = 0.779) and a moderate-to-strong positive correlation with general self-efficacy (*r* = 0.601). General self-efficacy was also positively correlated with self-confidence (*r* = 0.616).

Table 2 summarized the goodness-of-fit of confirmatory factor analysis. It showed excellent overall fit of the three-factor measurement model to the observed data. The comparative fit index (CFI = 0.993) and Tucker–Lewis index (TLI = 0.992) both exceeded the recommended threshold of 0.95. The root mean square error of approximation (RMSEA = 0.062, 90% CI: 0.052–0.072) was within the acceptable range (<0.08). Similarly, the standardized root mean square residual (SRMR = 0.072) was below the recommended cutoff of 0.08. These fit indices indicated that the hypothesized three-factor measurement model provided a good overall representation of the observed data. However, assessment of the measurement model also indicated differences in measurement performance across constructs. Satisfaction and self-confidence demonstrated relatively strong standardized factor loadings and adequate average variance extracted (AVE), whereas general self-efficacy showed comparatively weaker factor loadings and an AVE below the conventional 0.50 threshold. Thus, although the overall measurement model demonstrated good global fit, the convergent validity of the general self-efficacy construct was comparatively limited and should be considered when interpreting the structural associations.

**Table 2:** Goodness-of-fit indices for the confirmatory factor analysis (CFA) measurement model.

Fit index	Value
*χ*^2^ (df)	398.56 (222)
Comparative Fit Index (CFI)	0.993
Tucker–Lewis Index (TLI)	0.992
Root Mean Square Error of Approximation (RMSEA, 90% CI)	0.062 (0.052–0.072)
Standardized Root Mean Square Residual (SRMR)	0.072

Discriminant validity was further examined using the heterotrait-monotrait ratio (HTMT). HTMT values were 0.777 for satisfaction and general self-efficacy, 0.770 for self-confidence and general self-efficacy, and 0.889 for satisfaction and self-confidence. Thus, discriminant validity was generally supported, particularly for the distinction of general self-efficacy from the other constructs. However, the relatively high HTMT value for satisfaction and self-confidence indicates that these two closely related constructs were not as clearly differentiated as the other construct pairs.

Supplementary Table S3 summarizes the results of the confirmatory factor analysis (CFA). The measurement model showed a good fit to the data [*χ*^2^(222) = 398.56, CFI = 0.993, TLI = 0.992, RMSEA = 0.062 [90% CI: 0.052–0.072], SRMR = 0.072]. Standardized factor loadings were consistently high for the satisfaction (0.801–0.878) and self-confidence (0.774–0.837) constructs, indicating strong relationships between the observed indicators and their respective latent variables. Factor loadings for the general self-efficacy construct ranged from 0.498 to 0.639. This shows acceptable item contributions overall, although some indicators exhibited relatively weaker loadings than those of the other constructs.

The results of multiple linear regression as summarized in Table 3. After adjusting several covariates, both general self-efficacy and self-confidence were found to be statistically significant. We found that higher general self-efficacy was significantly associated with higher satisfaction (*B* = 0.037, 95% CI: 0.02–0.06), while self-confidence showed stronger positive association with satisfaction (*B* = 0.623, SE = 0.061, 95% CI: 0.50–0.74). Male students reported slightly higher satisfaction than female students (*B* = 0.20, 95% CI: 0.03–0.37). We did not find significant association for other covariates like age group, academic year, GPA, number of simulation sessions, or reason for choosing nursing. The model explained 64.0% of the variance in simulation satisfaction (*R*^2^ = 0.640; adjusted *R*^2^ = 0.611) and was statistically significant overall [F(15, 186) = 22.01, *p* < 0.001].

**Table 3:** Multiple linear regression analysis of factors associated with simulation satisfaction (*N* = 202).

Predictor	*B*	SE	95% CI	*p*-value
Intercept	0.15	0.26	−0.36, 0.66	0.559
Age group (ref: 18–20)				
21–23	0.03	0.14	−0.25, 0.31	0.855
24–26	−0.00	0.20	−0.40, 0.39	0.982
Gender (ref: Female)				
Male	0.20	0.09	0.03, 0.37	0.022
Academic year (ref: First year)				
Second year	−0.04	0.16	−0.35, 0.27	0.812
Third year	0.06	0.18	−0.29, 0.41	0.740
Fourth year	−0.05	0.21	−0.46, 0.36	0.808
Internship	0.03	0.26	−0.48, 0.54	0.910
Grade point average (ref: <3.50)				
3.50–4.49	0.11	0.10	−0.09, 0.32	0.272
4.50–5.00	0.14	0.12	−0.10, 0.37	0.257
Simulation sessions (ref: 1–2)				
3–5	0.04	0.11	−0.18, 0.26	0.722
>5	0.06	0.18	−0.29, 0.41	0.731
Reason for choosing nursing (ref: Employment/Career)				
Personal motivation	0.10	0.08	−0.06, 0.27	0.218
External influence	−0.17	0.15	−0.47, 0.14	0.277
General self-efficacy (continuous)	0.04	0.01	0.02, 0.06	<0.001
Self-confidence (continuous)	0.62	0.06	0.50, 0.74	<0.001

Model statistics: *R*^2^ = 0.640, Adjusted *R*^2^ = 0.611, F(15, 186) = 22.01, *p* < 0.001.

The structural equation model demonstrated a good overall fit to the observed data (Table 4). The comparative fit index (CFI = 0.992) and Tucker–Lewis index (TLI = 0.993) both exceeded the recommended threshold of 0.95, indicating excellent comparative model fit. The root mean square error of approximation (RMSEA = 0.054, 90% CI: 0.044–0.063) was below the recommended cutoff of 0.06 and its confidence interval remained well below 0.08. Likewise, the standardized root mean square residual (SRMR = 0.074) was below the recommended threshold of 0.08, indicating satisfactory residual fit.

**Table 4:** Goodness-of-fit indices for the structural equation model.

Fit index	Value
*χ*^2^ (df)	451.11 (285)
CFI	0.992
TLI	0.993
RMSEA	0.054 (90% CI: 0.044–0.063)
SRMR	0.074

The standardized structural path coefficients among the latent constructs were summarized in Supplementary Table S4. General self-efficacy was a strong positive predictor of self-confidence (*β* = 0.715, *p* < 0.001), while a greater number of simulation sessions was also positively associated with self-confidence (*β* = 0.352, *p* = 0.002). In contrast, academic year showed a modest negative association with self-confidence (*β* = −0.233, *p* = 0.037), whereas GPA was not significantly associated (*p* = 0.587). Both self-confidence (*β* = 0.650, *p* < 0.001) and general self-efficacy (*β* = 0.249, *p* = 0.002) were significant positive predictors. However, simulation sessions, GPA, and academic year were not significantly associated with satisfaction (all *p* > 0.05). The findings suggest that self-confidence and general self-efficacy are the primary factors associated with satisfaction with simulation-based learning, with self-confidence serving as the strongest predictor.

In this study, structural equation modeling showed that general self-efficacy exerted both direct and indirect effects on simulation satisfaction through self-confidence (Table 5). Specifically, general self-efficacy was positively associated with self-confidence (standardized indirect pathway), which in turn was positively associated with satisfaction. The standardized indirect effect of general self-efficacy on satisfaction through self-confidence was significant (*β* = 0.465, *p* < 0.001). This indicates that higher levels of general self-efficacy were associated with greater satisfaction via increased self-confidence. A significant direct effect of general self-efficacy on satisfaction also remained after accounting for self-confidence (*β* = 0.249, *p* = 0.002), resulting in a significant total effect (*β* = 0.714, *p* < 0.001). These findings indicate that self-confidence partially mediated the relationship between general self-efficacy and simulation satisfaction. Bootstrap mediation analysis using 5,000 resamples confirmed these findings, yielding a significant average causal mediation effect (indirect association = 0.057, *p* < 0.001) and a significant average direct effect (ADE = 0.037, *p* < 0.001). The total effect was also statistically significant (0.094, *p* < 0.001), with approximately 60.9% (95% CI: 43.0%–81.5%) of the total effect of general self-efficacy on satisfaction being mediated through self-confidence. In the context of this cross-sectional study, these estimates represent statistical associations within the specified mediation model and should not be interpreted as causal effects.

**Table 5:** Direct, indirect, and total effects of general self-efficacy on satisfaction: structural equation modeling and bootstrap mediation analysis.

Analysis and effect	Estimate	SE	95% CI	*p*-value
Structural equation model (standardized effects)				
Direct effect (GSE → Satisfaction)	0.249	0.167	—	0.002
Indirect effect (GSE → Self-confidence → Satisfaction)	0.465	0.148	—	<0.001
Total effect	0.714	0.190	—	<0.001
Bootstrap mediation analysis (5,000 resamples)				
Average causal mediation effect (ACME)	0.057	—	0.038–0.078	<0.001
Average direct effect (ADE)	0.037	—	0.016–0.056	<0.001
Total effect	0.094	—	0.074–0.111	<0.001
Proportion mediated	60.9%	—	43.0%–81.5%	<0.001

SEM effects are presented as standardized estimates (*β*). Confidence intervals were not reported for the standardized estimates because the 95% confidence intervals generated by the estimation procedure correspond to the unstandardized coefficients and are therefore not directly comparable with the standardized estimates.

## 4 Discussion

### 4.1 Main findings

Our study assessed the associations among general self-efficacy, self-confidence, and satisfaction with simulation-based learning among undergraduate nursing students using complementary multiple linear regression and structural equation modeling. The findings showed that general self-efficacy was positively associated with self-confidence and satisfaction, while self-confidence emerged as the strongest correlate of satisfaction and the association was statistically consistent with an indirect association through self-confidence. In addition, students who reported greater exposure to simulation sessions exhibited higher levels of self-confidence, whereas academic year showed a modest inverse association with self-confidence.

### 4.2 Comparison to previous studies

The positive association between general self-efficacy and self-confidence in our findings are consistent with Bandura's Social Cognitive Theory, which proposes that individuals with stronger efficacy beliefs are more likely to approach challenging situations with confidence, persist when confronted with difficulties, and perceive themselves as capable of successfully performing required tasks (7). Within simulation-based learning, students possessing higher general self-efficacy may engage more actively in clinical scenarios, showed greater willingness to make clinical decisions, and experience less anxiety during simulated patient encounters (4, 5). These students are likely to develop greater confidence in their clinical abilities during simulation activities. This interpretation is supported by a systematic review of nursing education interventions, which concluded that educational strategies incorporating simulation, debriefing, and active learning consistently strengthen students' self-efficacy and confidence during the transition to professional practice (19).

Our findings further demonstrated that both general self-efficacy and self-confidence were significantly associated with greater satisfaction with simulation-based learning, although self-confidence exhibited the strongest association. These findings align with previous studies reporting that nursing students who feel more confident during simulation activities generally report greater satisfaction with their learning experiences (14). Students who feel capable of understanding clinical scenarios and successfully performing required tasks are more likely to perceive simulation as meaningful, engaging, and educationally valuable (20). Conversely, students who experience uncertainty regarding their abilities may focus more on performance-related concerns than on learning opportunities, thereby reducing overall satisfaction.

Importantly, our mediation analysis suggests that self-confidence partially explains the association between general self-efficacy and satisfaction. This indicates that students with stronger general efficacy beliefs also tended to report greater simulation-related self-confidence, which was in turn associated with greater satisfaction. Although the cross-sectional design precludes conclusions regarding temporal ordering or causality, this pattern is consistent with the theory-informed model proposed in the present study. The positive association between the number of simulation sessions attended and self-confidence further reinforces the educational value of repeated simulation exposure. This association may plausibly reflect greater familiarity with simulation-based learning activities and repeated opportunities to engage with clinical tasks. However, the specific educational characteristics of individual simulation sessions were not assessed in this study, and therefore mechanisms such as structured feedback, reflective debriefing, or repeated skills rehearsal cannot be established as explanations for the observed association. Repeated participation provides students with multiple opportunities to rehearse clinical skills, receive structured feedback, engage in reflective debriefing, and gradually reduce performance anxiety (21, 22). Through repeated practice, students become increasingly familiar with simulation environments and develop greater confidence in applying theoretical knowledge to realistic clinical situations. Recent systematic reviews similarly conclude that simulation-based education improves learners' confidence, clinical competence, and practical skills across diverse nursing specialties (23).

Interestingly, academic year showed a modest negative association with self-confidence. Students in later academic years may differ from junior students in their clinical exposure, learning experiences, expectations, or perceptions of their own competence, but these factors were not directly assessed in the present study (24). Greater clinical awareness may therefore result in more cautious self-assessments despite increasing objective competence. Because relatively few studies have examined academic year as an independent correlate of simulation confidence while simultaneously accounting for general self-efficacy, further investigation is needed. Further longitudinal research is needed to clarify how self-confidence changes across nursing education and which educational factors may contribute to these differences.

In our findings, the consistency between the multiple linear regression analyses and the structural equation model strengthens confidence in the robustness of the observed associations. Whereas multiple regression evaluated relationships among observed composite scores, structural equation modeling simultaneously accounted for measurement error by modelling latent constructs. The convergence of findings across these complementary analytical approaches suggests that the observed associations were not solely attributable to measurement characteristics of the individual scales. Furthermore, the excellent fit of the measurement and structural models supports the construct validity of the three-factor framework used in this study.

An important consideration in interpreting the structural findings is the comparatively weaker measurement performance of general self-efficacy and the substantial empirical overlap between satisfaction and simulation-related self-confidence. Although the three-factor model showed good overall fit, general self-efficacy had lower factor loadings and an AVE below the conventional threshold. This indicates limited convergent validity in the present sample. In contrast, the HTMT results provided generally acceptable evidence for distinguishing general self-efficacy from satisfaction and self-confidence. The higher HTMT value between satisfaction and self-confidence (0.889) suggests that these constructs, while conceptually distinct, share substantial variance. This finding is understandable given that both were assessed in relation to the same simulation-based learning experience, but it also indicates that their structural associations should be interpreted with some caution. Taken together, these measurement findings do not invalidate the structural model, but they suggest that the magnitude and precision of associations involving these constructs should be interpreted in light of their observed measurement characteristics.

Several limitations should be acknowledged. The cross-sectional design used in this study precludes conclusions regarding causality or temporal sequencing. Although the structural equation model specified theory-driven directional pathways, these pathways should be interpreted as associations rather than causal effects. Longitudinal and experimental studies are needed to establish temporal relationships among self-efficacy, self-confidence, and satisfaction. Although several important academic characteristics were considered, the analyses could not account for other potentially influential factors. Variables such as previous healthcare or clinical experience, personality traits (e.g., resilience or conscientiousness), intrinsic learning motivation, anxiety or stress levels, academic workload, learning styles, quality of simulation facilitation and debriefing, simulation fidelity, instructor expertise, peer interactions, and institutional learning resources were not measured. In addition, the number of simulation sessions did not capture differences in the content, complexity, or educational characteristics of individual sessions, which may have resulted in variation in the intensity of simulation exposure. These unmeasured variables may have confounded or modified the observed associations. The study was conducted at a single university, which may limit the generalizability of the findings to nursing students in other educational settings or healthcare systems. All variables were measured using self-reported questionnaires and may therefore be subject to recall bias and social desirability bias. Although the measurement model showed good overall fit, the General Self-Efficacy Scale demonstrated relatively weaker convergent validity, with an AVE below the conventional threshold and lower factor loadings than the satisfaction and self-confidence constructs. This measurement limitation may have influenced the strength and precision of the observed structural associations involving general self-efficacy. The finding should therefore be considered when interpreting the direct and indirect associations involving this construct.

## Data Availability

The raw data supporting the conclusions of this article will be made available by the authors, without undue reservation.
