# Supplementary material for: General self-efficacy, self-confidence, and satisfaction in simulation-based nursing education: a structural equation modeling and mediation analysis
Source: Front Med (Lausanne). 2026 Oct 1;13:1945224. doi: 10.3389/fmed.2026.1945224 (PMC13668751; doi:10.3389/fmed.2026.1945224)
Supplement: Supplementary file 1 [file Supplementaryfile1.docx]

**Supplementary Materials**

**General Self-Efficacy, Self-Confidence, and Satisfaction in Simulation-Based Nursing Education: A Structural Equation Modeling and Mediation Analysis**

Majed M. Aljabri^1*^, Bandar S. Alharbi^1^

^1^Community and Psychiatric Mental Health Nursing Department, College of Nursing, King Saud University, Riyadh 12375, Saudi Arabia

^*^ Corresponding author (Email: [amajad@ksu.edu.sa](mailto:amajad@ksu.edu.sa))

| Construct | No. of items | Cronbach's α | McDonald's ω | AVE |
| --- | --- | --- | --- | --- |
| Satisfaction | 5 | 0.89 | 0.88 | 0.70 |
| Self-confidence | 8 | 0.92 | 0.91 | 0.65 |
| General self-efficacy | 10 | 0.77 | 0.73 | 0.30 |

Table S1. Descriptive statistics and psychometric properties

Table S2. Correlations among study variables

| Variable | 1 | 2 | 3 |
| --- | --- | --- | --- |
| 1 Satisfaction | — |  |  |
| 2 Self-confidence | 0.779*** | — |  |
| 3 General self-efficacy | 0.601*** | 0.616*** | — |

***p < .001.

Table S3. Model Fit and Standardized Factor Loadings

| Panel A. Model Fit |  | Panel B. Standardized Factor Loadings |  |
| --- | --- | --- | --- |
| Index | **Value** | **Construct** | **Range** |
| Chi-square | 398.56 (222) | Satisfaction | 0.801–0.878 |
| CFI | 0.993 | Self-confidence | 0.774–0.837 |
| TLI | 0.992 | General self-efficacy | 0.498–0.639 |
| RMSEA (90% CI) | 0.062 (0.052–0.072) |  |  |
| SRMR | 0.072 |  |  |

Table S4. Structural relationships among latent constructs

| Outcome | Predictor | Standardized β | SE | z | *p* |
| --- | --- | --- | --- | --- | --- |
| Self-confidence | General self-efficacy | 0.715 | 0.147 | 7.46 | <0.001 |
|  | Simulation sessions | 0.352 | 0.231 | 3.06 | 0.002 |
|  | GPA | 0.040 | 0.169 | 0.54 | 0.587 |
|  | Academic year | −0.233 | 0.137 | −2.09 | 0.037 |
| Satisfaction | Self-confidence | 0.650 | 0.141 | 6.13 | <0.001 |
|  | General self-efficacy | 0.249 | 0.167 | 3.03 | 0.002 |
|  | Simulation sessions | 0.110 | 0.191 | 1.54 | 0.124 |
|  | GPA | 0.054 | 0.152 | 1.07 | 0.283 |
|  | Academic year | −0.050 | 0.114 | −0.71 | 0.478 |
